# Androgens Suppress Corticosteroid Binding Globulin in Male Mice, Affecting the Endocrine Stress Response

**DOI:** 10.1210/endocr/bqae119

**Published:** 2024-09-06

**Authors:** Vera Sommers, Max Gentenaar, Karel David, Nick Narinx, Vanessa Dubois, Jan Kroon, Frank Claessens, Onno C Meijer

**Affiliations:** Laboratory of Molecular Endocrinology, Department of Cellular and Molecular Medicine, KU Leuven, Campus Gasthuisberg ON1 Herestraat 49 - Box 901, 3000 Leuven, Belgium; Department of Internal Medicine, Division of Endocrinology, Leiden University Medical Center, PO Box 9600, 2300 RC Leiden, Netherlands; Einthoven Laboratory for Experimental Vascular Medicine, Leiden University Medical Center, PO Box 9600, 2300 RC Leiden, Netherlands; Department of Internal Medicine, Division of Endocrinology, Leiden University Medical Center, PO Box 9600, 2300 RC Leiden, Netherlands; Einthoven Laboratory for Experimental Vascular Medicine, Leiden University Medical Center, PO Box 9600, 2300 RC Leiden, Netherlands; Laboratory of Clinical and Experimental Endocrinology, Department of Chronic Diseases and Metabolism, KU Leuven, ON1bis Herestraat 49 - Box 902, 3000 Leuven, Belgium; Department of Endocrinology, University Hospitals Leuven, 30000 Leuven, Belgium; Laboratory of Clinical and Experimental Endocrinology, Department of Chronic Diseases and Metabolism, KU Leuven, ON1bis Herestraat 49 - Box 902, 3000 Leuven, Belgium; Department of Laboratory Medicine, University Hospitals Leuven, 3000 Leuven, Belgium; Laboratory of Basic and Translational Endocrinology, Department of Basic and Applied Medical Sciences, Ghent University, 9000 Ghent, Belgium; Department of Internal Medicine, Division of Endocrinology, Leiden University Medical Center, PO Box 9600, 2300 RC Leiden, Netherlands; Einthoven Laboratory for Experimental Vascular Medicine, Leiden University Medical Center, PO Box 9600, 2300 RC Leiden, Netherlands; Laboratory of Molecular Endocrinology, Department of Cellular and Molecular Medicine, KU Leuven, Campus Gasthuisberg ON1 Herestraat 49 - Box 901, 3000 Leuven, Belgium; Department of Internal Medicine, Division of Endocrinology, Leiden University Medical Center, PO Box 9600, 2300 RC Leiden, Netherlands; Einthoven Laboratory for Experimental Vascular Medicine, Leiden University Medical Center, PO Box 9600, 2300 RC Leiden, Netherlands

**Keywords:** hypothalamus, pituitary, adrenal gland, glucocorticoids, androgens, androgen receptor

## Abstract

Biological sex affects the activity of the hypothalamus-pituitary-adrenal (HPA) axis. However, how androgen deprivation affects this axis remains largely unknown. In this study, we investigated the effect of androgen status on different components of the HPA axis in male mice. Two weeks of androgen deprivation did not affect total plasma corticosterone levels but led to increased pituitary ACTH levels. Stress-induced total plasma corticosterone levels were increased, whereas the suppression of corticosterone after dexamethasone treatment under basal conditions was attenuated. Androgen-deprived mice displayed a 2-fold increase in plasma levels of corticosteroid binding globulin (CBG). A similar increase in CBG was observed in global androgen receptor knock-out animals, compared to wild-type littermates. Androgen deprivation was associated with a 6-fold increase in CBG mRNA in the liver and enhanced transcriptional activity at CBG regulatory regions, as evidenced by increased H3K27 acetylation. We propose that the induction of CBG as a consequence of androgen deprivation, together with the unaltered total corticosterone levels, results in lower free corticosterone levels in plasma. This is further supported by mRNA levels of androgen-independent GR target genes in the liver. The reduction in negative feedback on the HPA axis under basal condition would suffice to explain the enhanced stress reactivity after androgen deprivation. Overall, our data demonstrate that, in mice, tonic androgen receptor activation affects CBG levels in conjunction with effects on gene expression and HPA-axis reactivity.

The hypothalamic-pituitary-adrenal (HPA) axis plays a vital role in the maintenance of physiological homeostasis and controlling the production of glucocorticoids in both a circadian manner and in response to stress. Activation of the HPA axis starts with the production of corticotropin-releasing hormone in the hypothalamus that acts on the pituitary gland, which in turn releases ACTH. Stimulated by ACTH, the adrenal glands secrete glucocorticoids into the circulation, primarily cortisol in humans, and exclusively corticosterone in mice. Following this release, glucocorticoids facilitate their own return to baseline levels via negative feedback mechanisms at the level of the pituitary gland and hypothalamus (1). Dysregulation of the HPA axis can contribute to several pathologies including immunodeficiency, diabetes, and mood and cardiometabolic disorders (1-4).

Glucocorticoids can affect the hypothalamus-pituitary-gonadal axis, in which hypothalamic GnRH regulates sex steroid production by the gonads via pituitary FSH and LH (5, 6). In turn, sex hormones regulate HPA axis activity (7, 8). For example, female rats display a higher increase in plasma corticosterone levels after restraint stress compared to male rats (9). Sex hormones not only influence the HPA axis activity directly, but also affect corticosteroid binding globulin (CBG) levels, a plasma protein that regulates the bioavailability of glucocorticoids (10). According to the free hormone hypothesis, only the unbound bioavailable fraction can exert an effect on the target tissues (11). In postpubertal female rats, plasma CBG levels were 2-fold higher compared to males, which was attributed to differences in GH levels that emerge during puberty (12). There is also strong evidence that estrogens regulate CBG levels both in rodents and humans because a reduction in CBG was observed in women with low estrogen levels (13), whereas during pregnancy and when taking the oral contraceptives, both estrogen and CBG levels rise (14). CBG production also increased in male rats upon estradiol administration (15).

These studies altogether demonstrate a role for estrogens in regulating HPA axis function and CBG levels, yet knowledge about the effects of androgens on these parameters is scarce. In this study, we investigated the effect of androgen status on the HPA axis function in male mice.

## Materials and Methods

### Animal Care

Mice were group-housed (2-4 mice/cage) under a constant temperature of 20 °C with a 12-hour light/dark cycle (light on 7 Am; lights off 7 Pm) and ad libitum access to water and standard chow diet. The animal experiments were conducted following the KU Leuven guidelines for animal experimentation and approved by the KU Leuven ethical committee (P139/2022 and P190/2020) or the Leiden University Medical Center ethical committee (PE.18.001.015) under the license number AVD1160020171084 granted by the Central Authority for Scientific Procedures on Animals complying with both the Dutch Act on Animal Experimentation and EU Directive 2010/63/EU.

### Experimental Design

#### Degarelix cohort

To study if androgen deprivation influences the HPA axis, 14-week-old male wild-type (WT) mice (C57BL/6J background) (Charles River, MA, USA) were chemically castrated using the GnRH antagonist degarelix (DGX) as detailed in the next section. At the moment of castration (day 0), mice were supplemented with either vehicle (VEH) or DHT for 2 weeks. Control mice (SHAM) received a subcutaneous injection with sterile H_2_O and were supplemented with VEH (details in section on hormone administration). All animals received analgesia with meloxicam (5 mg/kg) (Metacam, Boehringer Ingelheim, Ingelheim am Rhein, Germany) after the surgical procedure. For baseline measurements, rapid sampling via tail bleeding was performed to collected stress-minimized blood on day 7 at 18 Pm and on day 8 at 8 Am On day 8, mice underwent a novelty-stress test, and on day 11 a dexamethasone (DEX) suppression test was performed (more details in the section on HPA axis tests). Half of the mice were euthanized at day 14 by CO_2_ asphyxiation followed by cardiac puncture. Wet weights of androgen-sensitive organs were determined, and the hypothalamus, pituitary, adrenals, and liver were snap-frozen in liquid nitrogen and stored at −80 °C until further processing. The remaining mice were single-housed in metabolic cages for 24 hours to collect urine, which was stored at −20 °C to measure free urinary corticosterone levels, which reflect the free plasma levels given that protein-bound steroids do not enter the primary urine. Afterwards, mice were euthanized by CO_2_ asphyxiation followed by cardiac puncture, and the aforementioned organs were collected.

#### Orchidectomy and androgen receptor knock-out cohorts

In the orchidectomy (ORX) cohort, 14-week-old WT mice (C57BL/6J background) (Charles River) were surgically castrated via ORX. At moment of castration, mice were supplemented with VEH or DHT for 2 weeks. In the androgen receptor (AR) knock-out (ARKO) cohort, 14-week-old male global ARKO mice and WT littermates (C57BL/6J background) (16) received VEH for 2 weeks. All mice were euthanized at day 14 (ie, 16 weeks of age) by CO_2_ asphyxiation followed by cardiac puncture. Liver tissue was collected, snap-frozen, and stored by −80 °C for further processing.

### Castration Procedure and Hormone Supplementation

Chemical castration using the GnRH antagonist DGX was performed under isoflurane anesthesia (3% for induction, 2% for maintenance). Mice were subcutaneously injected with 25 mg/kg DGX (Ferring Pharmaceuticals, Saint-Prex, Switzerland) dissolved in sterile H_2_O (17). SHAM mice received a subcutaneous injection with sterile H_2_O. At the moment of castration, mice were subcutaneously implanted with a silastic stick in the dorsal region. VEH mice received an empty 1.5-cm silastic stick (Silclear Tubing; Degania Silicone) and DHT mice received a 1.5-cm silastic stick filled with 10-mg crystalline DHT (Fluka), resulting in a release of 75 µg DHT/day (18).

### HPA Axis Tests

#### Novelty-stress test

Stress-minimized blood via rapid tail bleed sampling was collected at timepoint 0 (8 Am). Subsequently, mice were subjected to a stress-inducing event by being transferred individually to a new cage without bedding for a duration of 10 minutes. After the 10-minute period, animals were placed back into their home cage and rapid tail bleed samples were taken at 10-, 30-, 60-, 90-, and 120-minute timepoint (19).

#### Dexamethasone suppression test

Mice were subcutaneously injected between 12 and 12:30 Pm with either 0.1 mg/kg dexamethasone phosphate (US Pharmacopeia) dissolved in PBS (DEX-injected) or PBS (VEH-injected) as control. After 6 hours, stress-minimized blood via rapid tail bleed sampling was collected (20).

### Corticosterone Measurements

Total corticosterone levels in plasma and free corticosterone levels in urine were measured with the Corticosterone HS (High Sensitivity) EIA kit (Immunodiagnostic Systems, Cat# AC-15F1, RRID: AB_3431967) according to the manufacturer's instructions. Plasma samples were diluted 25× and urine samples were diluted 50×. Relative free corticosterone levels in serum were calculated based on the Coolens formula (21), for which adjustments were made to the input variables. Indeed, the Coolens formula typically requires concentrations of both CBG and total corticosterone. In this study, CBG levels were measured as relative values through western blot analysis, whereas the concentrations of total corticosterone in serum were converted to relative values by normalizing these concentrations against the highest observed concentration of total corticosterone. The Coolens formula was then adjusted to use these relative values of CBG and normalized total corticosterone concentrations to calculate the relative free levels of corticosterone.

### ACTH Measurements

Snap-frozen pituitaries were homogenized in 50 µL RIPA buffer (Thermofisher). Protein concentration was measured in supernatant using a BCA kit (Pierce, Rockford, IL). To ensure uniformity all samples were diluted to a total protein concentration of 1 µg/µL, after which samples were diluted 10,000× to measure ACTH protein levels with the Mouse/Rat ACTH Elisa Kit (Abcam, Cat# ab263880, RRID:AB_2910221) according to the manufacturer's instructions.

### CBG Measurements

Livers were homogenized in RIPA buffer (1X PBS containing 1% IGEPAL [Sigma-Aldrich], 0.5% sodium deoxycholate, 0.1% SDS) and incubated on ice for 15 minutes. After centrifugation, protein concentration was measured in supernatant using a BCA kit (Pierce, Rockford, IL). Plasma was diluted 300× in PBS. Proteins were denatured by adding lithiumdodecylsulfate (Invitrogen) and Reducing agent (Invitrogen) at 70 °C for 10 minutes, separated on a NuPAGE Novex 4%-12% Bis-Tris Gel (Invitrogen), and blotted onto a polyvinylidene fluoride membrane (Amersham, Cytiva, Germany). Membranes were blocked with 5% nonfat dry milk in 0.1% PBS with Tween-20 at room temperature for 2 hours. Incubation with a primary antibody occurred at 4 °C overnight. The following antibodies were used: polyclonal rabbit anti-mouse CBG antiserum (The University of British Columbia Cat# Hammond_mouseCBGab, RRID:AB_2921648, kind gift of Prof. Geoffrey Hammond), anti-vinculin (Sigma-Aldrich Cat# V9131, RRID:AB_477629). Immunodetection was performed with LI-COR Odyssey XF using the Western Lightning Plus/Ultra-ECL reagent (Perkin Elmer), after incubation with horseradish peroxidase-conjugated secondary antibodies (Dako) at room temperature for 1 hour.

### Quantitative-real Time PCR

Hypothalamus, pituitary, and livers collected at sacrifice were snap-frozen and stored at −80 °C for further processing. Total RNA was extracted using Trizol reagent (Invitrogen) according to manufacturer's instructions. DNA was synthesized from 0.5 µg RNA (hypothalamus or pituitary) or 1 µg RNA (liver) using the FastGene Scriptase ready mix kit (NIPPON Genetics Europe, Dueren, Germany). The PCR reactions were performed using Fast SYBR Green Master Mix and the StepOnePlus Real-Time PCR system (Applied Biosystems, Foster City, CA, USA) Gene expression was normalized to the expression of the *18S RNA* (hypothalamus or pituitary) or *Hprt* (liver) housekeeping gene and expressed relative to the control group (2^−ΔΔCt^ method). Primers used are described in Supplementary Table S1 (22). All primers were designed to hybridize to different exons, and generation of single correct amplicons was confirmed by melting curve dissociation.

### Chromatin Immunoprecipitation

Livers were used for chromatin immunoprecipitation (ChIP) and processed as previously described (23). Livers were cut into small pieces in ice-cold PBS and pressed through a 70-µm cell strainer followed by a few passages through 18G and 21G needles. The homogenate was fixed with 1% formaldehyde at room temperature for 10 minutes followed by a 10-minute incubation with 125 mM glycine. After a wash with ice-cold PBS containing 1X PIC (Roche), cells were resuspended in lysis buffer (50 mM Tris-HCL pH 8.0, 10 mM EDTA, 1% SDS, and 1X PIC) and sonicated for 15 minutes (15 cycles 30 seconds on/30 seconds off using Bioruptor NGS from Diagenode). Chromatin (50 µg for H3K27ac ChIP) was diluted 10× in RIPA buffer (1X PBS containing 1% IGEPAL, 0.5% sodium deoxycholate, 0.1% SDS, and 1X PIC) and incubated overnight at 4 °C with 2 µg of H3K27ac antibody (Active Motif Cat# 39685, RRID:AB_2793305). The next day, Magna ChIP Protein A Magnetic Beads (Sigma-Aldrich), preincubated overnight at 4 °C with 5 mg/mL BSA and 40 µg/mL yeast tRNA, were added during 4 hours at 4 °C in the presence of 70 µg/mL yeast tRNA. Beads were washed 4 times with LiCL IP Wash Buffer (100 mM Tris pH7.5, 500 mM LiCl, 1% IGEPAL, and 1% sodium deoxycholate) containing 10 µg/mL yeast tRNA and 2 times with TE buffer (10 mM Tris-HCL pH 8.0, 1 mM EDTA). DNA was then eluted in 100 mM NaHCO_3_ containing 1% SDS and incubated overnight at 65 °C in the presence of 20 µg/mL proteinase K (Qiagen) for reverse-crosslinking. DNA purification was performed using the MinElute PCR Purification Kit (Qiagen, #2800), and samples were subjected to quantitative PCR analyses. The primer sequences are described in Supplementary Table S1 (22).

### Statistical Analysis

Statistical analysis was performed using GraphPad Prism V10.1.2 (GraphPad, La Jolla, CA, USA). Data are represented as mean ± SD. To compare 2 groups (WT vs ARKO), an unpaired Student *t*-test was performed. To determine difference between the 3 groups in the DGX or ORX experiment, a 1-way ANOVA with Tukey multiple-comparison test was used. Two-way ANOVA with Tukey multiple-comparison test was used in experiments with more than 1 independent variable. *P* < .05 were considered statistically significant.

## Results

### Validation of Androgen Deprivation and DHT Supplementation

**Figure 1.**
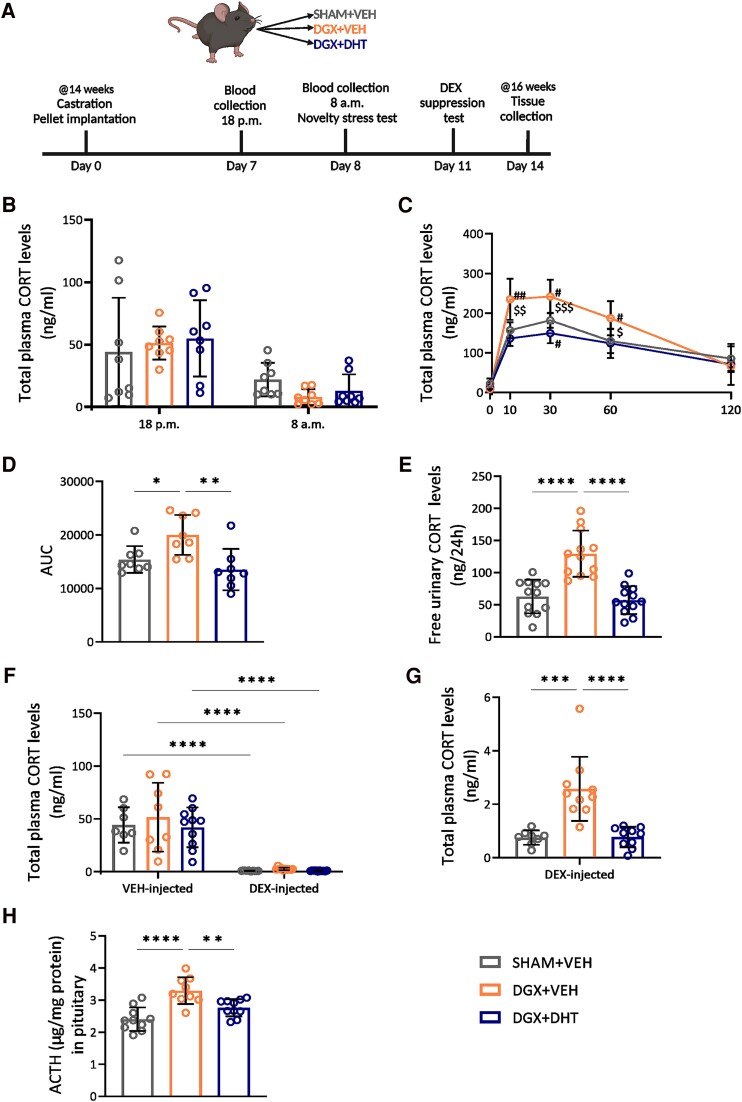
Androgen deprivation influences the stress-responses. (A) Experimental design. Fourteen-week-old male WT mice were chemically castrated with degarelix (DGX) and supplemented for 2 weeks with either vehicle (VEH) or dihydrotestosterone (DHT). The control mice (SHAM) received an injection with aqua ad iniectabilia and were supplemented for 2 weeks with VEH. (B) Total plasma corticosterone (CORT) levels at 6 Pm and 8 Am. (C-D) Time course of total plasma CORT levels after a novelty-stress event (C) and the corresponding area under the curve (AUC) (D). (E) Free CORT levels in urine after 24 hours in metabolic cages. (F-G) Total plasma CORT levels 6 hours after injection with VEH or 0.1 mg/kg DEX (F). Expanded axis of the DEX-induced levels from panel F (G). (H) ACTH levels in pituitary at day 14. N = 8-10/group. Data are represented as mean ± SD and were analyzed with 2-way (B, C, F) or one-way (D, E, G, H) ANOVA followed by Tukey multiple comparison test. *P < .05, **P < .01, ***P < .001, ****P < .0001; ^#^*P* < .05, ^##^*P* < .01 vs SHAM + VEH, ^$^*P* < .05, ^$$^*P* < .05, ^$$$^*P* < .001 vs DGX + DHT.

To determine the effects of androgen deprivation on the HPA axis, adult male WT mice were chemically castrated with DGX and compared with control animals (SHAM) and to castrated animals supplemented with DHT (DGX + DHT) (Fig. 1A). Androgen deprivation was confirmed by a reduction in the weight of the androgen-sensitive seminal vesicles (17), levator ani-bulbocavernosus muscle (24), and kidneys (25). Weight of all of these organs were restored by DHT supplementation (Supplementary Fig. S1A-C) (22). Altogether, these data confirm that the interventions were effective.

### Androgen Deprivation Affects the HPA Axis Function

Androgen deprivation did not influence total plasma corticosterone levels in the morning (8 Am) nor in the evening (6 Pm) (Figure 1B). Upon a novelty-stressor, total plasma corticosterone levels increased in the control animals (SHAM + VEH) as expected. This stress-induced increase in total corticosterone levels was higher in the DGX + VEH animals and remained higher up to 60 minutes compared to the SHAM + VEH animals, resulting in a higher area under the curve (Fig. 1C and D). Supplementation with DHT prevented this increase by DGX in stress-induced corticosterone levels (Fig. 1C and D). Next, we looked at urinary free corticosterone levels to investigate if the total increase in corticosterone upon stress reflects the urinary free fraction, given that single-housing in metabolic cages constitutes a substantially strong stressor (26, 27). DGX + VEH animals showed a 2-fold increase in free corticosterone levels in urine over a 24-hour period during our metabolic cage measurements (Fig. 1E). In addition, we assessed the effect of androgen deprivation on the negative feedback of the HPA axis by injecting 0.1 mg/kg DEX. As expected, total plasma corticosterone levels decreased after injection with DEX in all 3 groups (VEH vs DEX) (Fig. 1F). We observed that DGX-treated animals displayed a smaller reduction in corticosterone levels and that this was counteracted by DHT supplementation (Fig. 1G). Interestingly, after 14 days of intervention, an increase in pituitary ACTH protein levels was observed in DGX-treated animals, which was prevented by DHT supplementation (Fig. 1H). Altogether, these data indicate that androgen deprivation enhances the stress response of the HPA axis upon novelty-stress and attenuates the negative feedback loop in the HPA axis at the level of the pituitary. To establish whether changes in HPA axis response were due to changes in expression of the AR or the glucocorticoid receptor, we measured these via quantitative PCR analysis. Androgen deprivation did not influence expression levels of *Ar (Nr3c4)* and *Gr (Nr3c1)* in the hypothalamus and the pituitary (Supplementary Fig. S1D-E) (22).

### Androgen Deprivation Induces CBG Levels

**Figure 2.**
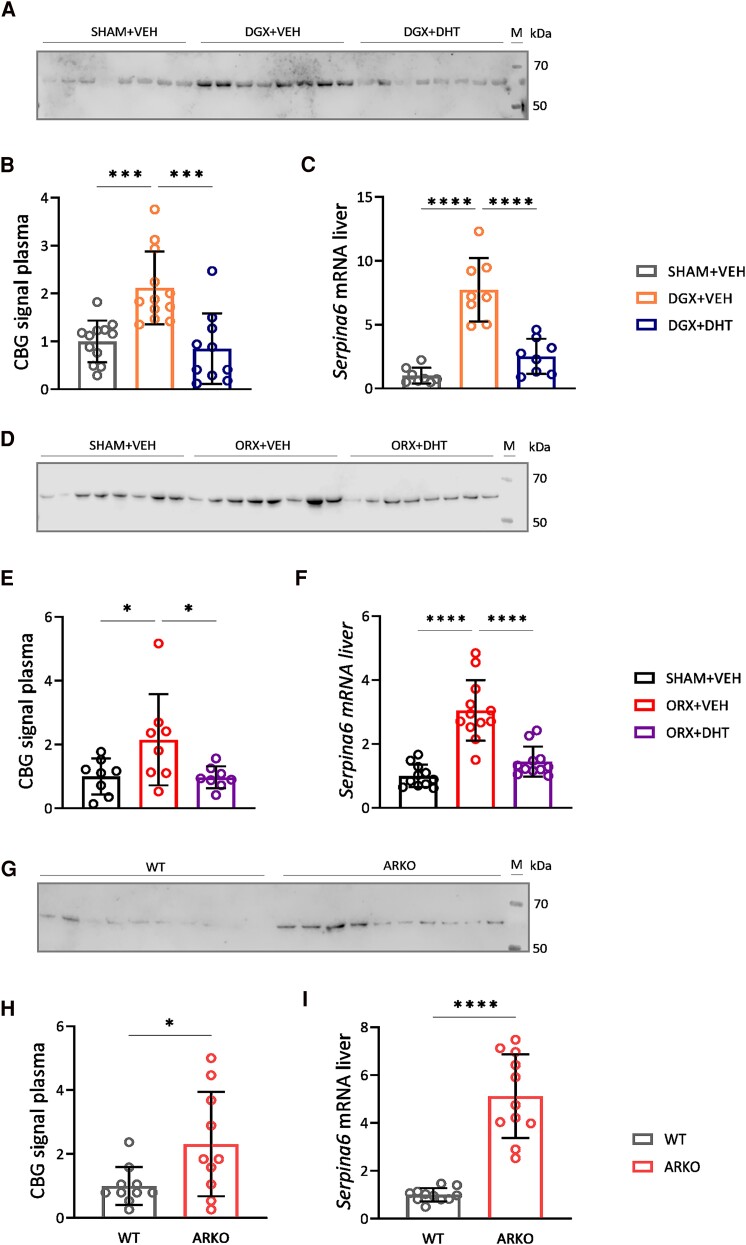
Androgen deprivation increases CBG levels. (A-I) Immunoblot of CBG in plasma, quantification of the immunoblot and relative mRNA expression of *Serpina6* encoding CBG in liver in the DGX model (A-C), the ORX model (D-F), and the ARKO model (G-I). N = 8-10/group. Data are represented as mean ± SD and were analyzed with 1-way ANOVA followed by Tukey multiple comparison test (B, C, E, F) or unpaired *t*-test (H, I). *P < .05, ***P < .001, ****P < .0001.

Bioavailability of corticosterone is regulated by CBG. We therefore measured CBG plasma levels and hepatic expression of the *Serpina6* gene. We observed a 2-fold increase of CBG plasma levels in the DGX-treated animals and a robust increase of *Serpina6* mRNA in the liver. Both these changes were prevented by DHT supplementation (Fig. 2A-C, Supplementary Fig. S2A) (22). The effect of androgens on suppressing CBG expression was confirmed in additional models of androgen deficiency (ie, castration by ORX (Fig. 2D-F, Supplementary Fig. S2B) and in ARKO mice (Fig. 2G-I, Supplementary Fig. S2C) (22). Because we observed increased expression of *Serpina6* in the liver of DGX-treated animals, we measured CBG protein levels in liver as well. However, we did not observe differences in either glycosylated or unglycosylated levels of CBG in the different treatment groups in the DGX or ORX models (Supplementary Fig. S3A and B) (22). Altogether, these data show that even though total basal plasma corticosterone levels were not affected by the different treatments, androgen deprivation increased plasma CBG levels.

**Figure 3.**
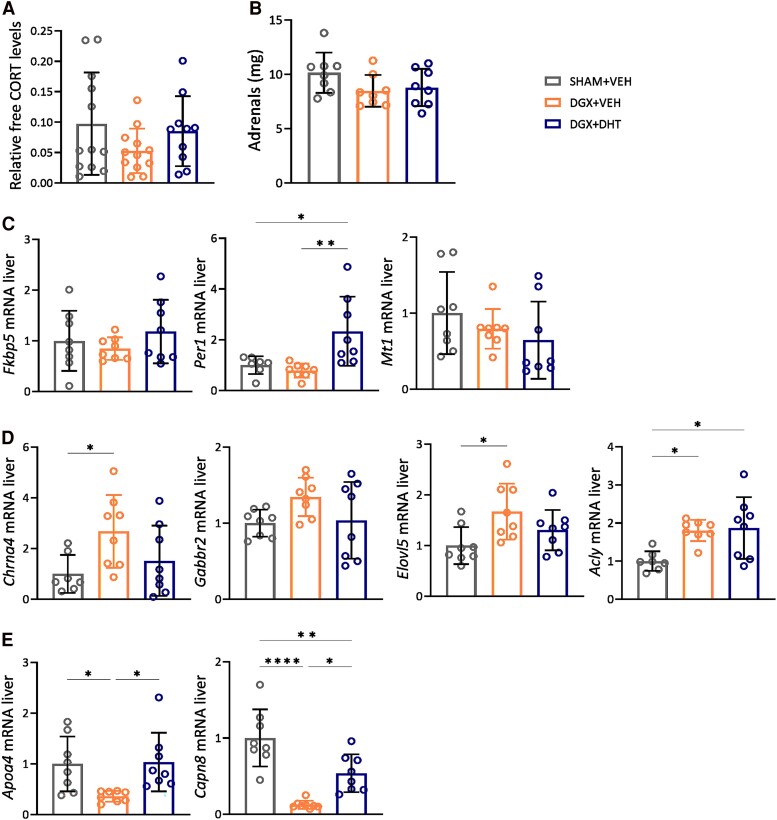
Androgen deprivation leads to lower predicted free CORT levels and changes in expression of GR target genes. (A) Relative levels of free corticosterone in plasma, calculated based on the Coolens formula. (B) Weight (at day 14) of the adrenal glands. (C-E) Relative mRNA expression of canonical GR target genes (C), AR-independent GR-repressed genes (D), and AR-independent GR-induced genes (E). N = 12/group. Data are represented as mean ± SD and were analyzed with 1-way ANOVA followed by Tukey multiple comparison test. *P < .05, **P < .01, ****P < .0001.

Upon androgen deprivation, free corticosterone levels were predicted to be lower compared to SHAM animals, based on total corticosterone levels and relative differences in CBG (Fig. 3A). Adrenal weight, which is sensitive to circulating corticosterone levels (28), was evaluated to reflect the potential effect of the lower free corticosterone levels. No significant differences in adrenal weight were observed between the experimental groups (Fig. 3B). Furthermore, we investigated whether or not the predicted lower free corticosterone levels affected GR activity in the liver by measuring the expression of the canonical GR target genes *Fkpb5, Per1,* and *Mt1.* There were no changes in expression of those 3 genes, except for an increase in *Per1* levels in the DGX + DHT group (Fig. 3C). It is important to note that these genes are both glucocorticoid and androgen responsive (29-31). We therefore additionally assessed the expression of GR target genes that are regulated independently of androgen status (32), namely the GR-repressed genes *Chrna4, Gabbr2, Elovl5,* and *Acly*, and the GR-induced genes *Apoa4* and *Capn8*. We observed a significant increase in the expression of *Chrna4, Elov5,* and *Acly* in the DGX + VEH group and supplementation with DHT prevented this effect for all genes with the exception of *Acly* (Fig. 3D). DGX + VEH animals showed a decreased hepatic expression of *Apoa4* and *Capn8*, which was prevented in the DGX + DHT group (Fig. 3E). This indicates that androgen deprivation modulates the expression of several AR-independent GR target genes, presumably via altering the availability of free corticosterone.

**Figure 4.**
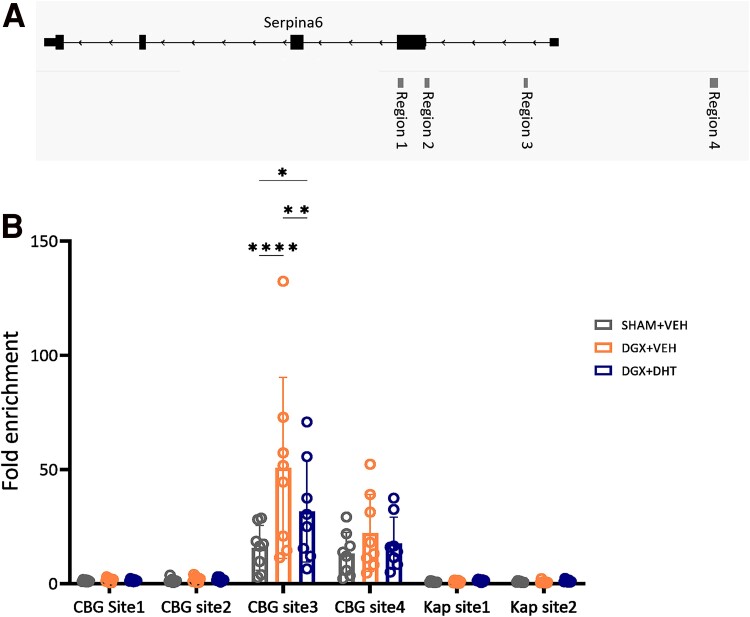
Androgen deprivation increases transcriptional activity around the CBG gene. (A) Schematic overview of the 4 regulatory regions around the CBG gene. (B) Levels of the H3K27ac histone mark at regulatory regions of the *Serpina6* and *Kap* (androgen-regulated in kidney but not in liver and therefore used as negative control) genes were assessed by ChIP-qPCR in livers after 14 days of treatment and are expressed as fold enrichments over the mean H3K27ac levels at 2 chromatin regions with no transcriptional activity. N = 8/group. Data are represented as mean ± SD and were analyzed with 2-way ANOVA followed by Tukey multiple comparison test. **P < .01, ****P < .0001.

To further decipher the mechanism behind the androgen effect on the hepatic transcription of the *Serpina6* gene, encoding for CBG, we investigated the impact of androgen deprivation on the transcriptional activity at regulatory regions using H3K27ac as epigenetic marker of active enhancers (33). Based on previously published liver ChIP data (34), 4 regulatory regions of the *Serpina6* gene were selected for further analysis (Fig. 4A). In the liver, androgen deprivation increased the active H3k27ac mark at regions 3 and 4, which were lowered by DHT supplementation (Fig. 4B, Supplementary Fig. S4) (22). This finding is in line with our observations that liver mRNA levels of the *Serpina6* gene are increased after androgen deprivation (Fig. 2B and D). As expected, neither androgen deprivation nor supplementation with DHT influenced the chromatin status at the regulatory regions of the kidney-specific *Kap* gene in these liver extracts (Fig. 4B).

## Discussion

**Figure 5.**
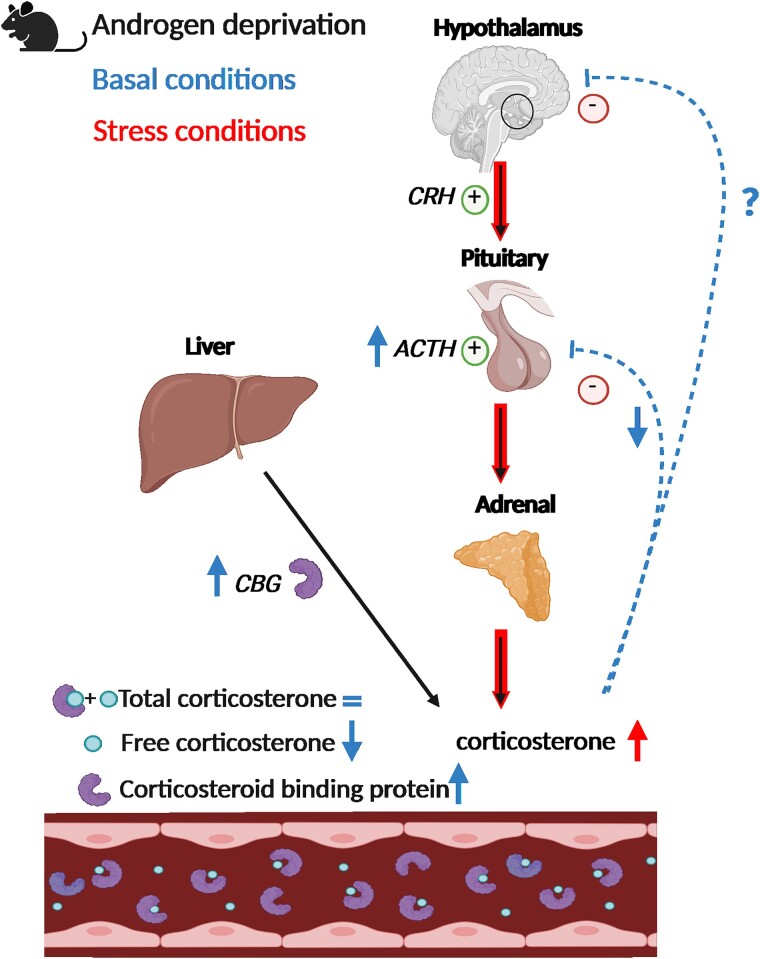
Overview of the impact of androgen deprivation on the HPA axis response, along with plasma CORT and CBG levels. Under normal conditions, androgen deprivation in male mice did not affect total plasma CORT levels in circulation but increased plasma CBG levels, leading to lower predicted free CORT (PFC). This would suffice to explain changes in mRNA and peptide expression in liver and pituitary, respectively. In addition, HPA axis sensitivity to a low dose of dexamethasone was diminished and ACTH protein levels in the pituitary were increased upon androgen-deprivation. Under stress, HPA axis reactivity was potentiated upon androgen deprivation. Created with BioRender.com.

In this study, we set out to unravel whether and how androgen status influences HPA axis function in male mice. We found no effects of androgen deprivation on corticosterone levels, but did observe that lower androgen status led to an increased HPA axis response to stress in association with higher pituitary ACTH levels, attenuated negative feedback under basal conditions, and an increase in basal plasma CBG levels (Fig. 5). These effects were AR-dependent and can be largely explained by lower free corticosterone levels as a consequence of increased plasma CBG (Fig. 2). The changes in the expression of AR-independent GR target genes in the liver further support a reduced bioavailability of corticosterone upon androgen deprivation (Fig. 3). However, we cannot assert with certainty that the observed effects were the consequence of changes in CBG and bioavailability of corticosterone.

We observed that 2 weeks of androgen deprivation did not change basal levels of total corticosterone but enhanced total corticosterone release after 10 minutes of novelty-stress. This effect was androgen-dependent because it was prevented by supplementation with DHT. Our findings are in line with a previous study that showed ORX enhanced the release of CORT after stress in male rats (35). In addition, several studies showed an inhibitory effect of testosterone on stress-induced corticosterone release in humans and rodents (36, 37). In trans-males, in which testosterone levels were increased after 6 months, no changes were observed in basal total cortisol levels, but ACTH-induced salivary cortisol levels were strongly blunted compared to baseline (36). In a study in male rats, higher levels of testosterone were shown to reduce corticosterone release in response to restraint stress (37). Our data extend these findings and additionally demonstrate that these effects are AR-dependent.

The corticosterone stress response involves both extensive neuronal processing of the stressor, and endocrine regulatory processes within the HPA axis (38). In relation to androgen effects on HPA axis reactivity, our data are compatible with a predominantly endocrine mechanism that revolves around CBG levels before onset of the stress. Based on the free hormone hypothesis, long-term elevated CBG leads to a smaller free fraction of hormone and lower exposure of target tissues to corticosterone (11, 39). Negative feedback within the HPA axis is predicted to be attenuated under conditions of reduced free corticosterone. This is in line with several studies suggesting weaker negative feedback in females compared to males because of higher levels of CBG in females (40, 41). Indeed, in our direct measure for negative feedback, we observed a modest but significant attenuation from dexamethasone suppression in castrated males. The increased pituitary ACTH levels suggest lower GR activation upon removal of androgens under nonstress conditions (42). The effects of basal androgen/free corticosterone concentration on ACTH content in the pituitary may result in similar stress-induced free corticosterone levels in males compared to females, but we were unable to get reliable data on the predicted free corticosterone concentrations in stress-conditions. This prediction is further complicated by the fact that CBG may be released from the liver in response to some stressors, making it impossible to extrapolate basal liver CBG content to stress-induced CBG concentrations in the blood (43).

We hypothesize that tonically reduced negative feedback via increased ACTH led to an increased capacity of the pituitary to respond to stress. A reduced negative feedback by the stress-induced corticosterone is predicted to prolong/enhance the HPA axis response to stress (44, 45), but this was not apparent from the time course after the acute and transient stressor of novel environment (Fig. 1C). The similar weights of the adrenal gland (Fig. 3B) in fact suggest that the effects of increased CBG concentrations on the HPA axis only become manifest under stressed conditions. The increased corticosterone response to stress extended to free corticosterone levels, as evidenced by the urinary free corticosterone levels (46) that were increased in DGX animals after 24 hours in metabolic cages. This setting is considered a significant stressor and is associated with increased sympathetic activation and corticosterone release over extended periods (26, 27). We cannot exclude that altered occupancy of GR or direct effects via AR in brain circuits involved in the stress response contributed to the changes in the HPA response as a consequence of the changed androgen status (44, 47).

A human genome-wide association study links the CBG locus to basal cortisol levels (48). The reduction in predicted free corticosterone upon androgen deprivation did not affect the basal corticosterone levels in our study, and this is in line with the data from earlier studies (36, 37). Within our experimental setup, we are unable to detect if circadian rhythm of corticosterone is influenced by androgen status. Our data are in line with a similar stress-induced HPA axis response reported in heterozygous CBG knockout mice that show a normal circadian rhythm of glucocorticoids (49).

We showed in 2 different castration models (ORX and DGX) and in an ARKO model that androgen deficiency increases the production of CBG in the liver resulting in higher plasma levels of CBG. This rules out a role of the pituitary gonadotropins FSH and LH because these respond in an opposite manner to ORX and DGX with increased gonadotropin levels upon ORX and decreased levels upon DGX (50). Several studies investigated the influence of sex hormones on the production of CBG both in vivo and in vitro (37, 51-53). These studies revealed conflicting observations on the effects of testosterone and DHT on CBG production, as Viau and Meaney reported that elevated levels of testosterone lowered CBG levels in male rats (37), whereas other studies reported no effect of androgens on CBG levels (51-53). It is important to note that the discrepancies related to androgen regulation of CBG can in part be explained by species differences and/or the specific conditions of studies. For example, the study of Smith and Hammond used androgen supplementation in gonadally intact rats (53). Studies therefore not always reflect how normal variations in testosterone levels regulate CBG levels. It was hypothesized that changes in CBG are explained via the effect of pubertal androgens on GH signaling, but in our study we did not investigate this. The finding that in mice with feminized AR high testosterone similarly enhanced HPA axis responsiveness (54) suggests a more direct role for the AR.

We found that androgen signaling regulates CBG at the transcriptional level in the liver. Our H3K27ac ChIP data indeed suggest that androgen deprivation increases transcriptional activity at regulatory sites 3 and 4 of the CBG gene. We were not able to get reliable ChIP signal with AR antibodies in this tissue. Nevertheless, it is notable that both these regulatory sites are binding sites for HNF4α (55) and STAT5 (56), which were not found in regions 1 and 2, suggestive of a possible interaction with these factors. HNF4α and STAT5 were previously shown to be a pioneer factor for AR (57, 58), meaning that these transcription factors can bind to condensed chromatin and initiate chromatin remodeling, making the DNA accessible for other transcriptions factors such as the AR. In prostate cancer cells, similar interactions between AR and STAT5a/b signaling were shown (59) and in hepatocellular cancer, CBG expression is regulated via the pioneer factors FOXA1 and the estrogen receptor, potentially underlying sex differences in CBG (60). Whether and how these observations connect to our findings of increased transcriptional activity in region 3 and 4 of the CBG gene in the absence of androgens and hence absence of active AR signaling requires further investigation.

In conclusion, we show that androgen deprivation increases plasma CBG levels in an AR-dependent manner, in association with increased transcriptional activity at regulatory regions of the CBG gene in the liver of androgen-deprived animals. In combination with unchanged total corticosterone levels, this increase in plasma CBG results in lower predicted free corticosterone levels in androgen-deprived animals. We observed increased ACTH protein levels in the pituitary and attenuated negative feedback after treatment with dexamethasone in basal conditions, whereas upon stress, a higher release of corticosterone was observed. Altogether, these data give more insight on the effect of androgen deprivation and can help understand the underlying mechanisms in the development of stress-related side effects in patients undergoing androgen deprivation therapy (61).

## Data Availability

Data generated or analyzed during this study are included in this published article or in the data repository listed in References (22).

## Abbreviations

AR: androgen receptor
ARKO: androgen receptor knock-out
CBG: corticosteroid binding globulin
ChIP: chromatin immunoprecipitation
DEX: dexamethasone
DGX: degarelix
HPA: hypothalamic-pituitary-adrenal
ORX: orchidectomy
VEH: vehicle
WT: wild-type
